# Harnessing the Interactions of Wound Exudate Cells with Dressings Biomaterials for the Control and Prognosis of Healing Pathways

**DOI:** 10.3390/ph17091111

**Published:** 2024-08-23

**Authors:** Shirin Saberianpour, Gianluca Melotto, Lucy Redhead, Nadia Terrazzini, Jaqueline Rachel Forss, Matteo Santin

**Affiliations:** 1 Centre for Regenerative Medicine and Devices, University of Brighton, Huxley Building Lewes Road, Brighton BN2 4GJ, UK; s.saberianpour@brighton.ac.uk (S.S.); g.melotto@brighton.ac.uk (G.M.); l.redhead@brighton.ac.uk (L.R.); n.terrazzini@brighton.ac.uk (N.T.); j.forss@brighton.ac.uk (J.R.F.); 2 School of Applied Sciences, University of Brighton, Huxley Building Lewes Road, Brighton BN2 4GJ, UK; 3 School of Health and Sport Sciences, University of Brighton, Falmer Campus, Village Way, Brighton BN1 9PH, UK

**Keywords:** wound dressings, inflammatory cells, progenitor cells, biocompatibility, prognosis

## Abstract

The global socioeconomic challenge generated by wounds requires an understanding of healing and non-healing pathways in patients. Also, the interactions occurring between the wound dressing biomaterials with cells relevant to the healing process have not been sufficiently investigated, thus neglecting the role that wound dressing composition can play in healing. Through the study of six cases of acute surgical wounds, the present work analyses the early (24 h post-surgery) interactions of biochemical and cellular components with (i) Atrauman, a device made of knitted woven synthetic polymeric fibre when used as a primary dressing, and (ii) Melolin, a hydrocolloid engineered as two layers of synthetic and cellulose non-woven fibres when used as a secondary dressing. A pathway towards healing could be observed in those cases where endoglin-expressing cells and M2 macrophages were retained by Atrauman fibres at the interface with the wound bed. On the contrary, cases where the secondary dressing Melolin absorbed these cell phenotypes in its mesh resulted in a slower or deteriorating healing process. The data obtained indicate that a subtraction of progenitor cells by Melolin may impair the healing process and that the analysis of the retrieved wound dressings for biomarkers expressed by cells relevant to wound healing may become an additional tool to determine the patient’s prognosis.

## 1. Introduction

Wound healing is a unanimously recognised clinical and socioeconomic problem on a global scale [1]. Wounds are broadly categorised as acute or chronic, the former mostly resulting in healing, while the latter fails to repair for weeks, months or years, leading to a deterioration of patient quality of life and, in a significant number of cases, to amputations and death [2]. It is known that the healing process of both acute and chronic wounds can be affected by a range of patient comorbidities and by infections.

In response to this growing clinical need, a range of wound dressings have been made available that differ from each other in their chemical composition, engineering and presence of therapeutic compounds, such as, for example, antibacterial agents [3]. The main approaches to the development of these devices are (i) engineering solutions to protect the wound from traumas (e.g., dressing made of knitted polymeric fibres) and bacterial colonisation (i.e., an oxygen-permeable polymeric film barrier) or (ii) engineering one or more absorbent layers to absorb the exudate while keeping the wound moist by the same oxygen-permeable polymeric film preventing bacterial invasion of the wound [4]. In fact, wound moisture is widely considered a prerequisite to maintaining the appropriate environment for the wound-healing process. These dressings can be used as such or in combination. In the latter case, they are referred to as primary and secondary dressings, whereby the primary dressing is often a mechanically resilient, knitted dressing, and the secondary is a hydrogel or hydrocolloid able to absorb excesses of wound exudate [5]. Typical wound dressings used as primary and secondary devices are Atrauman and Melolin [6,7]. Atrauman is a non-medicated tulle dressing consisting of water-repellent polyester fibres impregnated with fatty acids and recommended for use in combination with a secondary dressing [6]. While the engineered polyethylene fibres provide protection to fragile wounds, the triglycerides impregnation ensures low adhesion, thus reducing their disruption [6]. Melolin is a multi-layered device recommended for moderate wound exudates, where a film of polyethylene terephthalate protects a two-layer absorbent component, including a mixture of cotton and polyacrylonitrile fibres combined with a layer of non-woven cellulose fabric [7]. These two types of dressings are among the most used devices alongside dressings made of either synthetic (e.g., polyacrylic acids, polymethyl methacrylates) or natural polymers (e.g., alginate, carboxymethylcellulose) [5].

Currently, clinicians choose the dressings and consider their combination according to accepted wound classifications and, in particular, to the type of wound exudate, i.e., limited, moderate or large exudate volumes [8,9,10]. In this framework, the lack of consistent clinical outcomes deriving from the use of these devices has prompted studies aiming at the assessment of wound dressing efficacy, these being mainly based on clinical parameters [11,12,13].

An analysis of the many case studies and randomised clinical trials published over a few decades clearly shows that few of these investigations have focussed on the potential effects that wound dressings, as any other medical implants in contact with body fluids, may have on the healing process [14,15]. Indeed, unlike other clinical areas where the biocompatibility of implants has been studied at the level of interactions between biochemical and cellular components of the body fluid with the surface of the implant, the technological development and clinical use of wound dressings appear to have neglected the process known as the ‘foreign body response to implants’ [16]. Such a concept is based on data showing that, upon contact with the body fluid, the physicochemical properties of any implanted biomaterial (e.g., hydrophilicity/hydrophobicity, electric charges) can lead to the adsorption of proteins and consequent change of their conformation that makes them recognised by the inflammatory cells as antigens and to the consequent protracted activation of inflammatory cells leading to a chronic inflammatory status and to either the lack of healing or the formation of scar tissue.

It is argued that the study of the interactions of these biochemical and cellular components with the wound dressing biomaterials is not only important to ascertain their biocompatibility and prompt the development of new devices, but also has a potential value in identifying new biological markers of healing or not healing that can assist clinicians in their assessment of wounds and in their consequent choice of the best treatment, in a way that is more accurate than the current clinical parameters [17].

A biological marker, also referred to as a biomarker, serves as an indicator of the condition of a biological system [18]. Historically, parameters such as the erythrocyte sedimentation rate (ESR), C-reactive protein and albumin levels have been employed to evaluate the possibility of recovery and the presence of infections. Studies have identified various cellular processes and signalling molecules linked to wound healing, and it has been suggested that the clinical assessment of these agents in wounds may enable the wound prognosis thus hinting at their potential utilisation as biomarkers [19,20,21,22,23,24]. TNF-α, interleukins (e.g., IL-1, IL-6) and growth factors (e.g., platelet-derived growth factor, PDGF-BB) are secreted by cells such as macrophages, neutrophils, fibroblasts and platelets [25,26,27]. Research has demonstrated that elevated levels of several types of cytokines, exceeding the normal range, are frequently detected in non-healing wounds [28]. Additionally, evidence suggests that the administration of growth factors such as PDGF may expedite the healing process for persistent, non-healing wounds [27]. Nevertheless, the identification of a biomarker or a panel of biomarkers that is, at the same time, reliable and suited to clinical use remains an unmet challenge. For example, IL-6 lacks a reliable, predictive value for both favourable and unfavourable outcomes [26]. Consequently, these factors are seldom utilised in the assessment of wounds. More recent advances in genomics, proteomics, and molecular pathology have unveiled numerous potential biomarkers of clinical importance (e.g., microRNAs, long non-coding RNAs, and circular RNAs), but the technically complex, time-consuming and relatively expensive character of these techniques make them not implementable in clinical settings [29]. Likewise, the adhesion and activation of inflammatory cells such as neutrophils and macrophages are known to be affected upon adhesion with medical implants [16]. Despite that studies of neutrophils and macrophage interactions with wound dressings have been widely advocated [30,31], the systematic study of their interactions with wound dressings with different physicochemical properties, their phenotypical changes and potential harnessing as biomarkers of wound healing have not been sufficiently explored.

The aim of this study is to demonstrate, through six cases of toenail-surgery acute wounds in subjects with no ascertained relevant comorbidities, that the contact of the wound exudate with the biomaterials of primary and secondary dressings with different physicochemical properties leads to their interactions with biomolecules and cells inducing changes that can either favour or impair the healing process. The study of these interactions and the changes they induce in biomolecules and cells in relation to the final clinical outcome also paves the way towards the identification of specific biomarkers of healing or non-healing thus making the retrieved wound dressings a potentially accurate and clinically suitable prognostic tool helping clinicians to implement appropriate treatments by anticipating the pathway entered by the healing process.

## 2. Results

The clinical assessment and biochemical and cellular analyses of each patient were examined individually and linked to the final clinical outcomes. Participants’ demographic data and initial clinical assessment are reported in Table 1.

All patients showed monofilament scores of 10 in both LF and RF, ruling out any significant sensory loss. The Doppler analysis revealed a biphasic RF DP, a triphasic RF PT, a biphasic LF DP, and a biphasic LF PT, denoting an absence of peripherical arterial disease.

**Figure 1:**
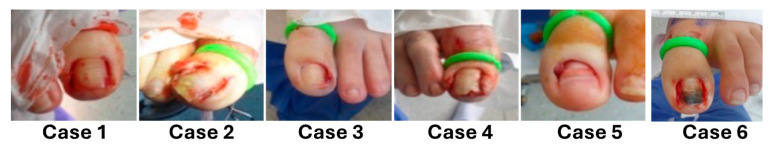
Participants’ wounds immediately after surgical procedures at Visit 2.

With the exception of Case 5, where a total nail avulsion was performed, all other patients underwent partial avulsion. Pre- and post-surgery wound areas were measured at each visit (Table 2), and healing progression evaluated as a percentage of the wound reduction or increase after the surgical procedure (Figure 1).

### 2.1. Case 1

A 20-year-old female with a BMI of 31.9, no history of diabetes or smoking and an IGTN of the fibular sulcus of the right hallux stage IIB (Table 1). At Visit 1, the patient’s wound was treated with Melolin as wound dressing, and she was recommended to regularly change the same dressing.

The patient returned five weeks later for surgery. At this stage, the wound did not show any sign of infection or of any other abnormal features, and a moderate exudate volume was observed. After surgery was performed (Figure 1, Case 1), primary and secondary dressings were applied. After one day, the patient returned for the final removal of the dressing prior to discharge with a wound area reduction of 17.6% (Table 2, Case 1).

**Figure 2:**
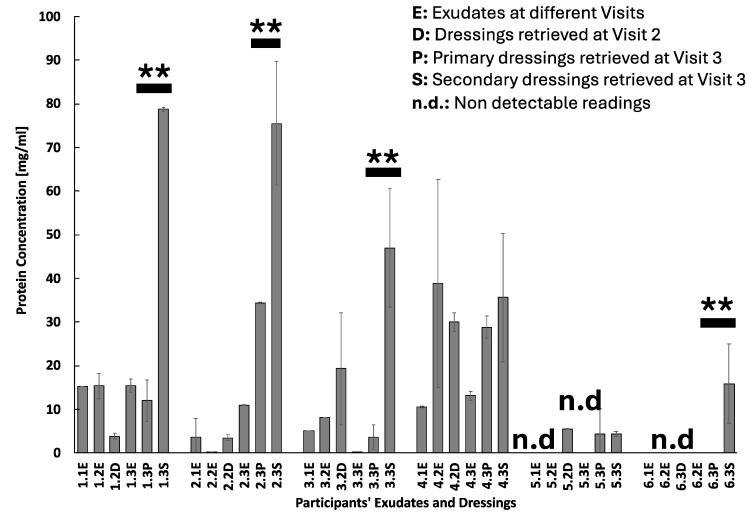
Protein concentration levels in exudates (E) and primary or secondary dressings (D, P, S) at different visits. Numbers indicate participant’s case study number and Visit number (e.g., 1.1E: Participant 1, Visit 1, Exudate). Statistical analysis was performed only on primary and secondary dressings protein levels at Visit 3. ** indicates statistically significant differences at *p* ≤ 0.01. Tests were performed by a Bradford assay and optical density values were converted into mg/mL through a standard curve obtained by measuring different concentrations of bovine serum albumin (BSA).

**Figure 3:**
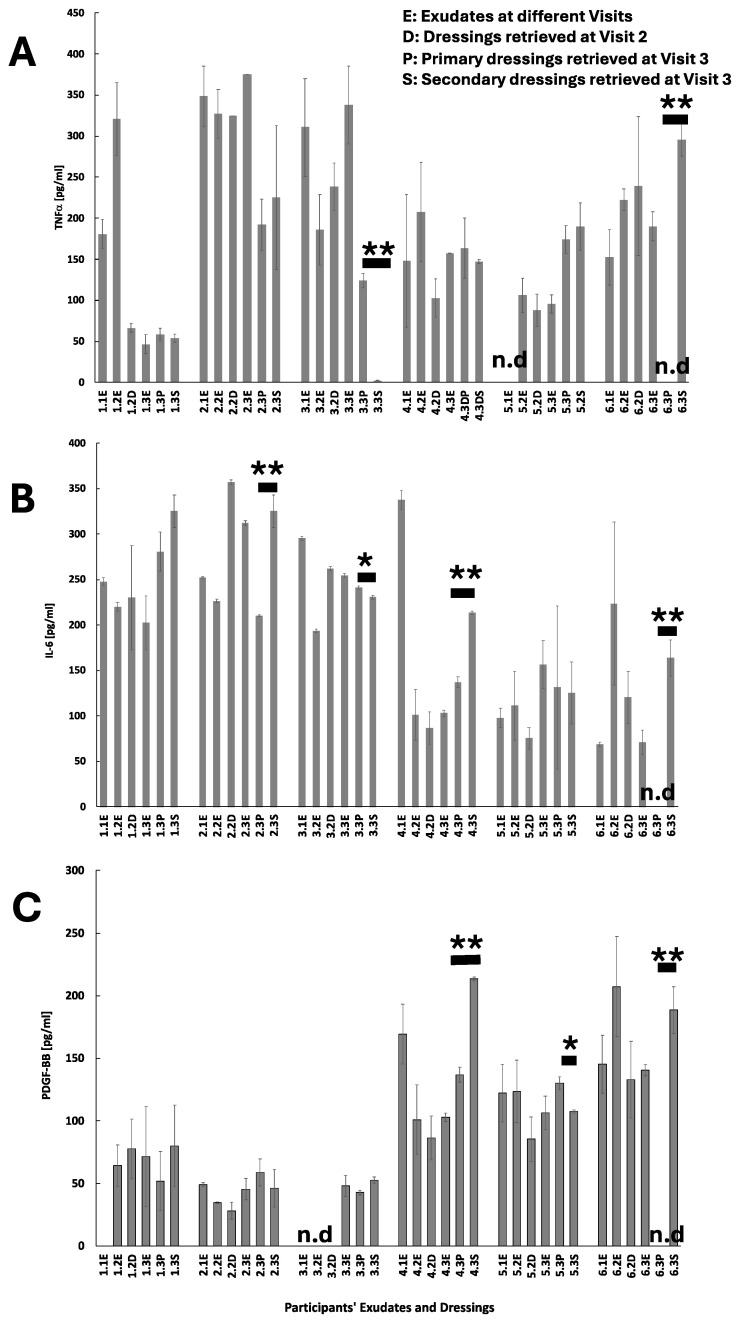
Participants’ levels of cytokines in exudates and retrieved dressings. (**A**) TNFα, (**B**) IL-6, (**C**) PDGF-BB. Statistical analysis was performed only on primary and secondary dressings cytokine and growth factor levels at Visit 3. * indicates statistically significant differences at *p* ≤ 0.05. ** indicates statistically significant differences at *p* ≤ 0.01. n.d. indicates not detectable values.

The overall protein concentration in the wound exudate was maintained constant across the three visits (ca. 15 mg/mL), but a gradual and sharp increase in the concentration of proteins absorbed by Melolin was observed with values ranging from 3.89 mg/mL in Visit 2 retrieved dressing to 78.80 mg/mL at Visit 3 retrieved dressing (Figure 2). The Atrauman applied at Visit 2 showed to have absorbed proteins at a concentration of 11.96 mg/mL (Figure 2). The analysis of the protein species, focussing on two cytokines relevant to acute inflammation (TNFα and IL-6), showed different concentration profiles (Figure 3A,B, Patient 1). In particular, the pro-inflammatory cytokine TNFα that was found at a concentration of 180.76 ± 18.02 pg/mL at Visit 1 reached a peak of 320.9 ± 44.59 pg/mL at Visit 2 and underwent a significant reduction of its concentration at Visit 3 46.51 ± 11.69 pg/mL (Figure 3A). Relatively lower concentrations of this pro-inflammatory factor were found in the Melolin absorbent layer at Visit 2 (66.46 ± 5.25 pg/mL) and Visit 3 (53.68 ± 5.08 pg/mL). Similar concentration levels were eluted from the primary dressing Atrauman at Visit 3 (58.20 ± 7.93 pg/mL). 

IL-6 profiles differed from those of the TNFα in both the exudate and dressings (Figure 3B, Patient 1). In particular, the concentration levels of this cytokine remained constant in the exudate and both the dressings across the visits with values at around 200 pg/mL, but for the concentration level found in Atrauman and Melolin at Visit 3, the concentration levels were 280.38 ± 21.84 pg/mL and 325.21 ± 17.96 pg/mL, respectively.

PDGF-BB levels in the exudates collected at Visit 2 and Visit 3 showed relatively low concentrations ranging from 64 to 71 pg/mL (Figure 3C, Patient 1). This factor seemed to be captured at similar concentration levels by both Atrauman (Visit 3: 51.95 ± 23.61 pg/mL) and Melolin (Visit 2: 77.72 ± 39.99 pg/mL, Visit 3: 80.06 ± 32.67 pg/mL).

**Figure 4:**
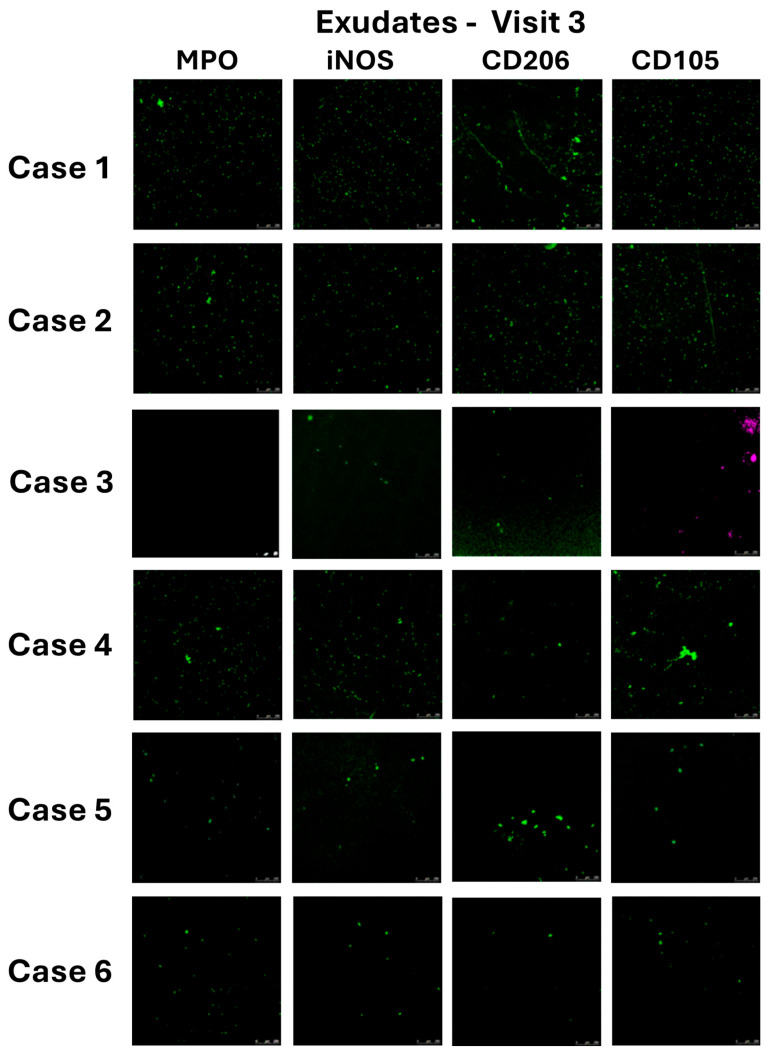
Immunostaining of patients’ exudates at Visit 3 (24 sh post-surgery). MPO: Neutrophil and platelet marker (myeloperoxidase), iNOS (nitric oxide synthase): M1 pro-inflammatory macrophages, CD206: M2 macrophage regenerative phenotype, CD105: progenitor cells. Scale bars = 100 μm. Wound exudates were diluted in PBS, applied to glass slides by smearing, fixed with paraformaldehyde, incubated with 3% *w*/*v* BSA blocking solution and finally stained with primary and fluorophore-tagged secondary antibodies.

**Figure 5:**
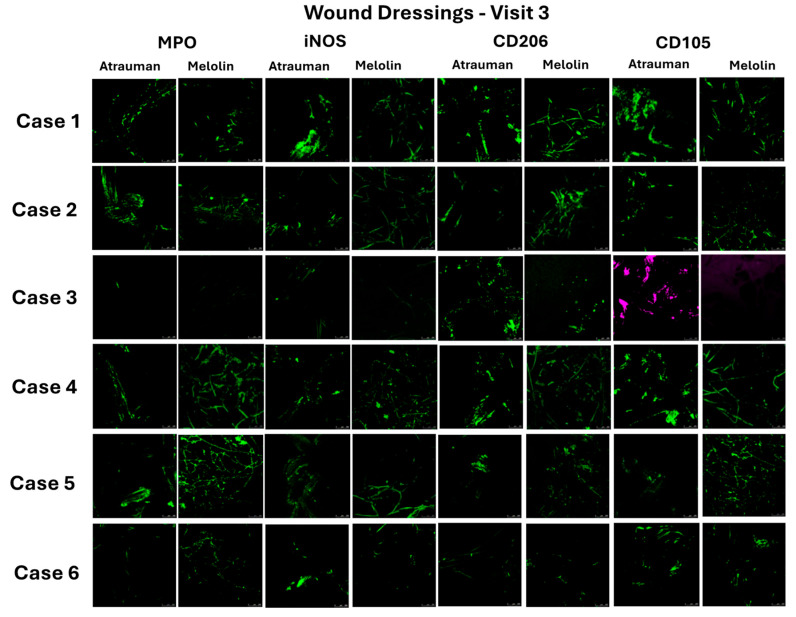
Immunostaining of patients’ primary and secondary wound dressings removed at visit 3 (24 h post-surgery). MPO: Neutrophil and platelet marker (myeloperoxidase), iNOS (nitric oxide synthase): M1 pro-inflammatory macrophages, CD206: M2 macrophage regenerative phenotype, CD105: progenitor cells. Case 3’s CD105^+^ cells were stained in purple to highlight the differences with all the other cases. Scale bars = 100 μm. Withdrawn dressings were copiously washed with PBS, fixed with paraformaldehyde, incubated with 3% *w*/*v* BSA blocking solution and finally stained with primary and fluorophore-tagged secondary antibodies.

This patient showed a very low cell count in the exudate of the first visit (12,353/100 μL) that gradually increased during the healing process to reach 1,494,000 cells/100 μL (Table 3). The immunostaining of the exudate at Visit 3 showed a uniform presence of all the cell phenotypes investigated (Figure 4, Case 1), while the analysis of the same phenotypes in contact with the primary and secondary dressing showed higher cell densities with cells forming aggregates, particularly in the case of their adhesion on the Atrauman fibres (Figure 5, Case 1).

### 2.2. Case 2

A 19-year-old male with a BMI of 24.5, a previous smoker with no history of diabetes and with an IGTN of the fibular sulcus of the right hallux stage IIB (Table 1) visited and treated with Melolin returned for surgery after 10 days. The wound after surgery (Figure 1, Case 2) was dressed with Atrauman as a primary wound dressing and Melolin as a secondary wound dressing. After one day, the patient returned to clinics for final treatment and discharge, showing a wound area reduction of 42.4% (Table 2).

This patient also showed a relatively low concentration of proteins in the exudate during the first two visits and an increase at levels similar to those of Case 1 at Visit 3 (11 mg/mL) (Figure 2). The values of protein concentrations absorbed by Melolin at Visits 2 and 3 were also consistent with the values of Case 1, but higher concentrations were measured in Atrauman (34.33 mg/mL) (Figure 2). This patient showed constantly higher levels of TNFα in the exudates of the three visits (>300 pg/mL, Figure 3A, Patient 2), with dressings absorbing levels of this pro-inflammatory cytokine relatively higher than in Case 1 (Figure 3A, Cases 1 and 2). In the case of IL-6, the concentrations were at levels similar to Case 1, but for the last visit, the Case 2 patient showed an increase in this cytokine (Figure 3B, Case 2). Melolin showed higher concentrations of IL-6 than Atrauman at Visit 3, the latter absorbing lower levels than those observed on the same biomaterial in Patient 1 (Figure 3B, 2.3P and 2.3S).

This patient showed concentration levels of PDGF-BB in all exudates and dressings similar to Case 1 (Figure 3C, Cases 1 and 2).

The number of cells in the exudate of this patient over the three visits sharply increased between Visits 1 and 2 to then undergo a relative decrease at Visit 3 (Table 3, Visit 1: 21,851 cells/100 μL; Visit 2: 766,451 cells/100 μL; Visit 3: 540,800 cells/100 μL). The immunohistochemistry of the exudates and retrieved dressings at Visit 3 were similar to that of Case 1, showing all phenotypes consistently observed (Figure 4 and Figure 5, Case 2).

### 2.3. Case 3

A 49-year-old female with a BMI of 28.4, a previous smoker with no history of diabetes and an IGTN of the fibular sulcus of left hallux stage IIA (Table 1) was treated with Melolin and returned for surgery after 3 days (Figure 1, Case 3). After one day, the patient returned to clinics for final medication and discharge, showing a reduction of the wound area of 63.9% (Table 2).

This patient showed low protein concentrations in the exudates collected during the three visits with a peak observed at Visit 2 (Visit 1: 5 mg/mL, Visit 2: 8 mg/mL, Visit 3: 0.05 mg/mL), concentration levels were absorbed by Melolin as per Case 1 and 2 (Visit 1: 19 mg/mL, Visit 2: 47 mg/mL), but lower protein concentrations were found in both Atrauman (3.64 mg/mL) and Melolin (ca. 48 mg/mL) at Visit 3 (Figure 2, Case 3).

In this patient, the relatively high levels of TNFα and IL-6 observed in the exudate at Visit 1 dropped significantly at Visit 2, where Melolin appeared to have absorbed concentrations of these two pro-inflammatory cytokines but returned to high values after surgery as their levels were higher at Visit 3 in the exudate and in the two dressings (Figure 3A,B, Case 3).

Noticeably, this patient showed non-detectable levels of PDGF-BB in the exudates of Visits 1 and 2, while the levels of this growth factor were similar to those of the other previous patients at Visit 3 (Figure 3C, Case 3).

Case 3 also showed very low levels of cell numbers in the exudate with a peak detected at Visit 2 (Table 3, Visit 1: 5468 cells/100 μL; Visit 2: 12,404 /100 μL; Visit 3: 5125/100 μL). Consistently, the characterisation of the cell subpopulations in the exudate of this patient at Visit 3 showed scarce traces of all the cell phenotypes with the exception of the CD105^+^ progenitor cells that appeared as both single cells and aggregates (Figure 4, Case 3). High levels of adhesion of this type of cells and of the CD206^+^ M2 macrophages were observed in the Atrauman retrieved at Visit 3, but no cell adhesion was detected in Melolin (Figure 5, Case 3).

### 2.4. Case 4

A 62-year-old male with a BMI of 28.1, no history of diabetes or smoking and an IGTN of both fibular and tibial sulcus of the right hallux stage III (Table 1) was treated with Melolin and returned for surgery after five weeks. After surgery (Figure 1, Case 4), Atrauman and Melolin were applied as primary and secondary dressings, and the patient returned to clinics the day after for final medication and discharge, showing a reduction of the wound area of 15% (Table 2). 

This patient had starting levels of proteins in the exudate at Visit 1 comparable to the previous cases, but they increased to values higher than Cases 1 to 3 at Visit 2 with a significant protein concentration being found in the Melolin (Figure 2, Case 4). After surgery, the levels of protein concentrations appeared to be relatively low in the exudate, but higher in both the primary and secondary dressing. The TNFα concentration remained at similar values in the exudates and dressings at all visits (Figure 3A, Patient 4). Conversely, IL-6 showed a high exudate concentration value at Visit 1 to then be significantly reduced at Visits 2 and 3 in both exudates and primary dressing, but increased in Melolin at Visit 3 as observed in Cases 1 and 2 (Figure 3B, Patient 4). 

In this patient, the concentration of PDGF-BB in the Visit 1 exudate was relatively higher than the other previous cases at Visit 1, but dropped at constantly lower levels in the exudates of Visits 2 and 3 (Figure 3C, Case 4). Noticeably, Melolin absorbed relatively high levels of this growth factor, similar to the previous cases at Visit 2, but it also showed relatively high levels in Atrauman (Figure 3C, Case 4).

The number of cells in the exudate of this patient was also relatively high since Visit 1 (210,370), peaking at Visit 2 (374,375) and registering a significant decrease at Visit 3 (137,666) (Table 3). The characterisation of the subpopulations in the exudate of Visit 3 showed a relatively high density of MPO^+^ neutrophils and iNOS^+^ M1 macrophages, with a scarce density of CD206^+^ M2 macrophages and a significant presence of CD105+ progenitor cells in the form of single cells and aggregates (Figure 4, Case 4). A similar pattern appeared in the dressings where neutrophils and M1 macrophages were mainly observed to establish contacts with Melolin and M2 and progenitor cells with Atrauman (Figure 5, Case 4).

### 2.5. Case 5

A 36-year-old female, with a BMI of 24.2 without a history of diabetes and smoking presented an IGTN of the proximal nail fold (retronychia) on the left hallux unstageable (Table 1), showing inflammation and low levels of drainage.

At Visit 1, Melolin was applied as wound dressing and the patient returned to the clinic for Visit 2 after 4 weeks to undergo a total nail avulsion of the nail with phenolisation of the nail matrix (Figure 1, Case 5). Atrauman and Melolin were applied as primary and secondary dressings, and the patient returned to clinics the day after for final medication and discharge showing an increase in the wound area of 38.9% (Table 2).

Noticeably, the exudates showed non-detectable levels of proteins and relatively low levels absorbed into Melolin at Visit 2 and into both Atrauman and Melolin at Visit 3 post-surgery (Figure 2, Case 5). The levels of TNFα in the exudate were also non-detectable at Visit 1, but they increased pre-surgery at Visits 2 and 3 and were absorbed at similar levels (ca. 100 pg/mL) in Melolin at Visit 2. At Visit 3 both Atrauman and Melolin absorbed similarly higher amounts (Figure 3A, Case 5). Wound exudates and dressings at all stages showed constant levels of IL-6 and PDGF-BB (ranges: 70 to 150 pg/mL, Figure 3B,C, Case 5), with Atrauman retaining levels of PDGF-BB significantly higher than Melolin at Visit 3, post-surgery (Figure 3C, Case 5).

This patient showed the highest exudate cell number at Visit 1, which underwent a decline at Visit 2 to return to levels similar to Visit 1 after surgery at Visit 3 (Table 3, Case 6). These exudates showed relatively low densities of all the cell phenotypes investigated (Figure 4, Case 5), but the analysis of the same subpopulations adhering to the primary and secondary dressings at Visit 3 showed a tendency of MPO+ neutrophils, CD206+ M2 macrophages and CD105+ progenitor cells to be adhering more on Melolin than Atrauman (Figure 5, Case 5).

### 2.6. Case 6

A 17-year-old female with a BMI of 18.8 without a history of diabetes or smoking presented an IGTN of the fibular sulcus of the left hallux stage IIB (Table 1).

On visit 1, Melolin was applied as wound dressing and the patient returned to the clinic for Visit 2 after 20 days to undergo a bilateral partial nail avulsion of the nail with phenolisation of the nail matrix. After the surgery, Atrauman and Melolin were applied as primary and secondary. The participant returned to the clinic 24 h after the surgery for a follow-up appointment showing a wound reduction of 5.4% (Table 2, Case 6). The wound bed was entirely granulating and serosanguineous exudate was observed. Erythema and swollen tissues were localised distally to the interphalangeal joint and no signs of infection were observed. The wound was redressed with Melolin and the participant discharged.

As per Case 5, the total protein concentration levels were also undetectable in all the exudates and dressing collected with the exception of the Melolin retrieved at Visit 3 (Figure 2, Case 6). This patient showed relatively high levels of TNFα, IL-6 and PDGF-BB concentrations in all samples with the exception of the Atrauman at Visit 3, where no detectable values were obtained (Figure 3A–C, Case 6).

The exudate cell numbers were reduced from Visit 1 to Visit 2, but sharply increased post-surgery at Visit 3 (Table 3, Case 6). In this exudate, the various subpopulations under investigation had a relatively low density (Figure 4, Case 6), but relatively higher levels of adhering MPO^+^ neutrophils and CD105^+^ progenitor cells were observed in Melolin (MPO^+^ and CD105^+^ cells) and Atrauman (CD105^+^ cells) (Figure 5, Case 6).

### 2.7. Protein Adsorption

**Figure 6:**
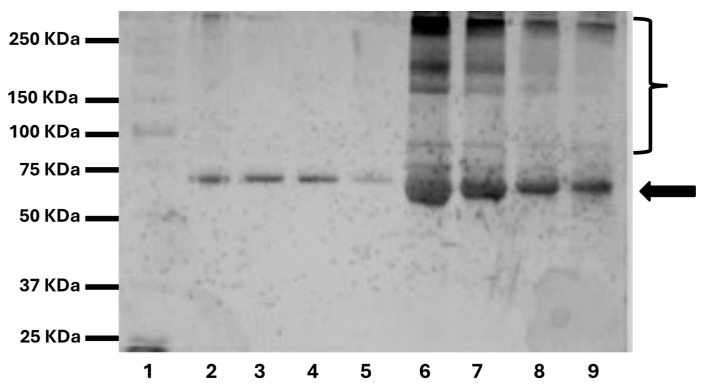
Protein elution profiles from different types of wound dressing. Lane 1: Molecular weight standards, Lanes 2 to 5: proteins eluted from Atrauman, Lanes 6 to 9: proteins eluted from Melolin. Lanes 2 and 6: proteins eluted by a 10% *v*/*v* isopropanol/water washing, Lanes 3 and 7: proteins eluted by a 30% *v*/*v* isopropanol/water washing, Lanes 4 and 8: proteins eluted by a 50% *v*/*v* isopropanol/water washing, Lanes 5 and 9: proteins eluted by a 70% *v*/*v* isopropanol/water washing. Arrow indicates a 68 kDa protein putatively identified as human serum albumin [32]. Bracket indicates a range of high-molecular-weight proteins putatively including cold fibronectin and immunoglobulins. Experiments were performed on the dressing of each patient providing reproducible results.

The gel electrophoresis of the eluted proteins clearly showed different patterns of adsorption for the two types of dressings. Atrauman led to the adsorption of only one main protein band that was putatively identified as a 68 kDa albumin (Figure 6, Lanes 2 to 5, black arrow) [32]. The peak of elution of this protein occurred upon incubation in 30% *v*/*v* and 50% *v*/*v* isopropanol/water media. In the case of Melolin, more protein species were eluted (Figure 6, Lanes 6 to 9, black arrow and bracket). Proteins in the molecular weight range of 250 kDa to 75 kDa are likely to include, among others, immunoglobulin, cold fibronectin and fibrinogen (Figure 6, Lanes 6 to 9, bracket) [32]. These were mainly released upon incubation with 10% *v*/*v* and 30% *v*/*v* isopropanol/water media, indicating that proteins were either loosely bound to the Melolin fibres or likely to be still in a soluble state entrapped within its mesh.

## 3. Discussion

While many studies have highlighted the role of specific cytokines, growth factors and cell phenotypes in acute and chronic wounds, no clear biochemical and cellular pathway of healing or non-healing has been unequivocally identified [25,26,27,28]. The main obstacles found by these investigations are likely to be related to the different types of wounds, patients’ health conditions and comorbidities, treatment protocols and analytical methods adopted.

At the same time, the effect of the wound dressings’ physicochemical properties and biocompatibility has been neglected despite many studies have demonstrated that the interactions occurring between proteins and cells with the surface of medical implants are inevitably altered, leading to the formation of a film of adsorbed proteins and to phenotypical changes in adhering inflammatory and tissue cells [16,32].

The present work was designed to study the interactions that the wound dressing biomaterials have with proteins and with cells present in the exudate. The aim was to find out whether these interactions could affect the healing process and be harnessed to establish and monitor potential biomarkers of healing or non-healing. It was hypothesised that the identification of any specific biochemical or cellular pattern either in the wound exudate or at the surface of the retrieved dressings would not only shed light on the therapeutic role or adverse effect of specific wound dressings on tissue repair, but it would also turn the retrieved dressing into a prognostic device.

To this end, the study was designed to analyse the effect that two types of dressings with different physicochemical compositions could have on acute wounds as those generated by a relatively limited surgery, ingrown toenail avulsions, on six patients with no relevant comorbidities.

To highlight the advantage of focussing on the wound dressing/protein/cell interactions rather than the wound bed, biochemical and phenotypical markers found in contact with the dressings were compared with those of the wound exudate. In addition, the clinical cases were selected on the basis of the combined use of the primary dressing, Atrauman and the secondary dressing Melolin when in contact with the wound exudate for a relatively short period of time, 24 h. Such a short implantation time enables the analysis of the early interactions between biochemical and cellular species with the dressing, thus emphasising any effect caused by the biomaterial surface physicochemical properties.

As expected, a glance at the clinical assessment did not show any clear link between the patients’ reported general clinical history and the 24 h healing response to the surgery. However, it was clear that according to the Martinez-Nova classification [33], Case 3 presented a less invasive nail fold/nail plate ratio (IIA) compared to Cases 1, 2 and 6 whose conditions were classified as IIB, to Case 4 who was classified as III and to Case 5 who was unstageable. Indeed, in Case 3, the surgical procedure caused a wound of a size (Visit 2, 29.6 mm^2^) comparable to that of Case 1 and significantly smaller than all the other cases, but the wound size in this patient was significantly reduced after only 24 h of treatment with Atrauman and Melolin. All the cases showing a reduction of the wound size were reported to have a reduction of the cell numbers in the exudate at Visit 3. The cases where wound healing did not appear to progress (Cases 1 and 6) or to regress (Case 5) showed increased cell numbers in the exudate. This clinical observation corresponded also to the ability of this patient’s wound to show low levels of cell numbers before and after surgery in the exudate with a population of CD105^+^ cells adhering in clusters, particularly at the surface of the Atrauman knitted fibres (Figure 5, Case 3, Atrauman, CD105) 24 h after surgery. In this case, there was no detectable CD105^+^-cell in the superimposed Melolin secondary dressing, indicating that these progenitor cells had not been subtracted from the wound bed [34,35]. This seemed to distinguish Case 3 from all the other cases. In particular, the cases with less wound reduction, while showing adhesion of CD105^+^ cells on Atrauman fibres, they revealed cell penetration across Atrauman into Melolin where they adhered on its fibres (Figure 5, Cases 1, 2 and 4, CD105). More noticeably, the worsening wound size conditions of Case 5 corresponded to a high level of penetration of CD105^+^-cells within the secondary Melolin dressing with no traces in the primary Atrauman dressing and in the exudate (Figure 4 and Figure 5, Case 5, CD105). These results seem to indicate that any absorption of exudate from the wound by the more hydrophilic Melolin secondary dressing has subtracted from the exudate cells playing an important role in the healing process, while the more hydrophobic Atrauman, as primary dressing in direct contact with the wound, has made the same cells available to the healing process at the interface with the wound bed. Likewise, Case 6 showed less penetration in the Melolin dressing and some levels of CD105^+^ cells on the Atrauman fibres (Figure 5, Case 6, CD105), but to a lesser extent than all the other cases except Case 5.

CD105 is a marker of endoglin, a transmembrane protein relevant to angiogenesis and wound healing, mostly expressed by endothelial cell progenitors [34], mesenchymal stem cells and fibroblasts. Its presence in wounds has been observed as early as two days after injury [35]. The present work demonstrates that, while only weak positive staining was observed in exudates 24 h after surgery, cells expressing this marker can be detected more reliably by analysing the retrieved wound dressings.

Case 3 clearly also showed a distinct profile of inflammatory cell interaction with the dressings when neutrophils (MPO^+^ cells) [36], M1 macrophages (iNOS^+^ cells) [37,38] and M2 macrophages (CD206^+^ cells) [39] were investigated (Figure 6, Case 3). In this case, no neutrophils or M1 macrophages were observed, but the regenerative M2 phenotype population appeared to be enriched at the wound bed/Atrauman interface.

Among all the other cases showing a relatively smaller wound reduction in 24 h, neutrophils (and to a lesser extent M1 macrophages) were observed and appeared in both the primary and secondary dressings, while in the case of the deteriorating Case 5 and not progressing Case 6, neutrophils were mainly absorbed by Melolin (Figure 5, MPO). These data, together with the absence of any significant CD206^+^ cell in the Atrauman dressing of Cases 5 and 6, seem to indicate a wound environment in a pro-inflammatory status rather than the activation of a regenerative pathway.

Alongside its different engineer of the fibres in a tulle rather than hydrogel structure, Atrauman appears to minimise protein adsorption limiting it to the adsorption of albumin (Figure 6, Lanes 2 to 5), a relatively hydrophobic protein known to ‘passivate’ biomaterials surface in terms of cell adhesion [16,32]. This can be explained by the presence of the impregnation of this type of dressing with relatively hydrophobic triglycerides [6]. Although this type of coating has been introduced by the manufacturer to reduce adherence of the dressings to the wound [6], the results of this study demonstrate that the coating also contributes to the albumin-driven passivation of the biomaterial surface thus explaining the relatively limited adhesion of cells on its fibres. On the contrary, Melolin was shown to absorb and adsorb more protein species among which the presumptive identification made in this study indicates the possible presence of ‘cold fibronectin’, IgG and fibrinogen (Figure 6, Lanes 6 to 9); proteins known to promote the adhesion and activation of platelets, inflammatory and tissue cells [32].

The insights provided by the present investigation are likely to be relevant also to the use of other types of wound dressings. Indeed, previous papers have compared the swelling properties of Melolin to other absorbent dressings including the carboxymethylcellulose-based devices Aquacel and Kerracel and the alginate-based Kaltostat [40,41]. In particular, Uzun et al. have reported a swelling of approximately 20% wt/wt in Kaltostat and 19% wt/wt in Aquacel [40], while 25% wt/wt in Kaltostat and 15% wt/wt in Aquacel were observed by Minsart et al. [41]. The percentage of Melolin swelling has been reported to be 18% wt/wt thus making its exudate absorbing potential similar to Kaltostat and Aquacel [40]. Therefore, although not used in the present study, it can be speculated that the effect of both Kaltostat and Aquacell when adopted as secondary dressings would lead to results similar to that observed with Melolin raising questions about the use of highly absorbent dressings in cases where a healing pathway is, as in the case of acute wounds, in progress. To fulfil the need to remove excessive exudate volumes while preserving a favourable cell population in the wound bed, absorbent dressings such as the carboxymethyl cellulose-based Kerracel should rather be used. In fact, while absorbing a relatively high volume of exudates, Kerracel is known to undergo a high swelling of its fibres capable of obstructing the penetration of the cells throughout its mesh [42].

When compared to the exudates where no clear links could be found between the total cell number (Table 3), cell subpopulations (Figure 4 and Figure 5) and wound healing, the profiles of these cell biomarkers in the wound dressings show the potential advantage that wound dressings can offer as tools to monitor healing processes. This was not necessarily the case for the studied biochemical markers where the only trend seems to be that the concentrations of the total protein content and of TNFα, IL-6 and PDGF-BB were higher in Melolin than Atrauman, post-surgery.

## 4. Materials and Methods

Clinical treatments. The study of 6 clinical cases of onychocryptosis, commonly referred to as an ingrown toenail (IGTN), treated with surgical excision and redressed with a combination of Melolin (Smith and Nephew, Watford, UK) and Atrauman (Hartmann Buckingham, UK) wound dressings was performed at the Leaf Hospital (University of Brighton, Eastbourne, UK). The experimental protocol was ethically approved by the University of Brighton Ethics Committee (Brighton Research Ethics Application Manager, Ref: 2023-11385-Forss) and the Health Research Authority Ethics Committee (Integrated Research Application System, Project ID: 318917). The ethics panels’ approval included the recruitment of patients from the age of 16 years. Patient Number 6 (17 years old) was recruited following the signing of the consent form by both the patient and parents. In addition, all researchers involved in the collection, storage, use and disposal of human samples received formal training to comply with the UK Human Tissue Act regulations. Eligibility criteria include subjects with an active IGTN with moderate levels of exudate and without clinical signs of infection. Participants for this study were recruited between April and July 2023. During the nail surgery assessment appointment (Visit 1) eligible subjects were approached by a dedicated research podiatrist. The participant information sheet was provided to eligible subjects. Following the appropriate time for consideration of the participant information sheet and discussion with the research podiatrist, consent to participate was gained through the completion of the consent form. Subjects who were unable to provide informed consent were excluded from the evaluation. Surgical treatments were individually agreed with participants and followed the best practice principles. Procedures taken at each visit are summarised in Table 4.

On Visit 1, participants’ demographic and baseline clinical data were collected alongside exudate sampling (Table 4). Participants were tested for peripheral arterial disease (PAD) and peripheral neuropathy (PN). PAD was assessed by analysing the posterior tibial (PT) and the dorsalis pedis (DP) artery of the left foot (LF) and the right foot (RF) with an 8MHz Doppler ultrasound. PN was assessed by analysing 10 areas of LF and RF with a 10-g Semmes-Weinstein monofilament. In addition, IGTNs were staged according to the onychocryptosis classification outlined by Martinez-Nova et al. [34] (Table 5).

On Visit 1, the IGTN exudate was collected using a swab (FLOQSwabs 520CS01, COPAN Diagnostics, Murrieta, CA, USA) and diluted in 0.5 mL of sterile phosphate buffer saline (PBS) pH 7.4. Melolin dressing was applied to the wound according to the wound management guidelines. Participants were advised to change the dressing regularly and to apply Melolin dressing 24 h before the surgery appointment (Visit 2).

On Visit 2, the Melolin wound dressing was removed, and two sections (1.5 cm × 1.5 cm) were cut where the exudate was absorbed. Each section was collected either in 5 mL of sterile PBS for cell studies or in sterile deionised water for protein adsorption studies. 

At this stage, surgery was performed by experienced podiatrists following the latest Royal College of Podiatry nail surgery guidelines. Participants underwent either partial or total nail avulsion under local anaesthetic with phenolisation of the nail matrix of the hallux. The procedure involved the following stages: after peri-surgical skin disinfection, a digital nerve block of the hallux was performed by injecting 3% *w*/*v* plain mepivacaine hydrochloride (Scandonest, Septodon, Maidstone, UK). A tourniquet was then applied to the base of the hallux and kept in place for the entire procedure (for a maximum of 20 min). After the partial or total removal of the toenail, 80% *v*/*v* liquified phenol (Phenol Swab-it, PodoPro, Huddersfield, UK) was applied twice for one minute to ablate the nail matrix and the tourniquet removed. Depending on the clinical needs, the surgery resulted in wounds of different sizes, as shown in Table 2, Column Visit 2.

The exudate sample was collected as described for Visit 1, after the removal of the nail, but prior to the phenol application. 

Following surgery, Atrauman was applied to the wound as the primary dressing, while Melolin was added as the secondary dressing. Participants were booked for a 24 h post-surgical follow up (Visit 3), as per clinical guidelines.

On Visit 3, both the primary and secondary dressings were removed, cut into two sections (1.5 cm × 1.5 cm), and collected in sterile PBS and deionised water as described for Visit 2. Subsequently, the exudate sample was collected as for Visits 1 and 2. Participants were finally medicated by applying a Mepore (Molnlycke Health Care, Oldham, UK) on the wound bed and discharged.

**Figure 7:**
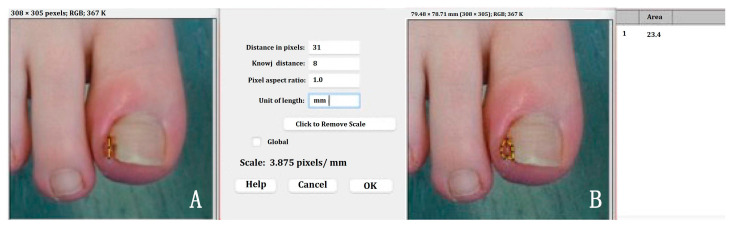
Wound images analysed by ImageJ software. (**A**) Software calibration was obtained using the function ‘straight line’ and inserting the known distance (i.e., wound length) manually measured during the visit. (**B**) Subsequently, the wound area was calculated after drawing the wound edges with the function ‘polygon sections’.

The maximum length and width of the wounds were measured by a research podiatrist by using a sterile paper ruler and photos were taken at every visit. The wound area was then calculated using ImageJ software, as elucidated in Figure 7A,B.

Biochemical and cellular studies. For biochemical and cellular experiments, the collected samples were transferred to the laboratories of the School of Applied Sciences and Centre for Regenerative Medicine and Devices, University of Brighton, within 2 h from collection.

Protein concentrations in both exudates and eluates of the dressings were measured by using Bradford’s assay (BioRad, Watford, UK), while TNFα, IL-6 and PDGF-BB concentrations were measured by using ELISA (Abcam, Cambridge, UK). The choice of these three factors was made on the basis of their widely accepted role in the healing process, whereby TNFα is involved in the early acute phase of inflammation, IL-6 contributes to the acute inflammatory process while playing a role in its transition to healing steps, and PDGF-BB is released by both activated platelets and macrophages considered the most potent inducer of fibroblast proliferation. 

Cells were separated from the exudates by centrifugation at 1000× *g* and counted by haemocytometer after resuspension in 100 µL of PBS and finally processed as smears for immunostaining by applying 10 μL on microscope glass slides. Three markers of inflammatory cells were studied: (i) myeloperoxidase (MPO) for neutrophils [35], (ii) CD68 for the monocytes/macrophages overall population [36], (iii) induced nitric oxide synthase (iNOS) for pro-inflammatory macrophage (M1) phenotype [37], (iv) CD206 for post-inflammatory macrophage M2 phenotype [38], and (v) CD105 for haemoprogenitor cells [34] by confocal microscopy. Immediately after smearing on the glass slides, cells were fixed in formalin for 10 min, washed three times with PBS and then treated for 1h with 3% *w*/*v* bovine serum albumin solution as blocking agent to eliminate any subsequent non-specific binding of the antibodies. After further washes in PBS, the samples were incubated in antibody (rabbit anti-human MPO, CD68, iNOS, CD206 and CD105 Abcam, Cambridge, UK) solution diluted 1:100 in the same blocking solution for 1 h, room temperature. Following the same washing procedures, a secondary FITC-conjugated goat anti-rabbit IgG antibody solution diluted 1:100 in the same blocking solution was added for 1 h at room temperature and in dark conditions. Confocal microscopy was performed on all smears and dressing samples at ×20 magnification.

The biochemical and cellular data were related to the adsorption of proteins on the dressing fibres where the binding strength was analysed by a previously published method [30]. Briefly, after 2 h of transportation, the incubation in sterile deionised water was established to be sufficient to remove the excess proteins entrapped in the dressing mesh, but not tightly bound to their fibres. At arrival, the excess water was removed from the dressings by contact with tissue paper. Dressings from each patient were then sequentially immersed in increasing concentrations of isopropanol/water solutions (IsoPOH/Water, 10%, 30%, 50%, 70% *v*/*v*) to detach proteins from the fibre surface according to their binding strength. The eluted proteins were freeze-dried overnight and then resuspended in 10 μL of electrophoresis sample buffer (BioRad, Watford, UK). Electrophoresis was performed according to a standard sodium dodecyl sulphate poly(acrylamide) gel electrophoresis (SDS-PAGE) method using a Mini Protean electrophoresis kit (BioRad). The electrophoresis was performed at 100 mV for 2 h. To enhance the detection of protein species bound to the fibres in low amounts, a sensitive silver staining (BioRad, Watford, UK) of the gels was adopted. Protein electrophoretic profiles were documented by image analyser by visible light (BioRad, Watford, UK).

Statistical analysis. Biochemical and cell data were analysed by ANOVA two-way test from n = 3 using GraphPad Prism version 8 software. Data were considered significantly different at *p* ≤ 0.05.

## 5. Conclusions

The current study provides evidence of the different interactions that biochemical and cellular components establish with Atrauman (a dressing manufactured in the form of woven knitted fibres made of a synthetic polymer) and Melolin (a hydrocolloid engineered as different layers of polyester and cellulose non-woven fibres). When the two dressings are combined as primary and secondary dressings, it appears that any condition (e.g., excessive exudate absorption by the hydrogel-based absorbent layer) leading to the removal of endoglin-expressing cells (CD105^+^) and/or M2 macrophages by absorption into the secondary dressing can impair healing. This appears to be the case when the hydrogel-based Melolin absorbs relatively high levels of these two types of cells in its mesh. At the same time, the predominance of adhesion of M2 macrophages and CD105^+^ cells with the knitted fibres of the primary dressing Atrauman was linked to a healing pathway. These findings also suggest that the rapid detection or absence of these two types of cells on the Atrauman fibres can be harnessed as a prognostic tool in healing/non-healing. However, to be adopted in clinics as prognostic tools, the tests of the interactions between cells and dressing will require further efforts to develop methods of detection of these cell phenotypes that are rapid, cost-effective and easy to interpret.

## Figures and Tables

**Table 1:** Participants’ demographic data and initial clinical assessment.

Patients	Age	Gender	Body Mass Index	Smoking	Diabetic	Martinez-Nova’s Classification Visit 1
Case 1	20	Female	31.9	No	No	IIB
Case 2	19	Male	24.5	No	No	IIB
Case 3	49	Female	28.4	No	No	IIA
Case 4	62	Male	28.1	No	No	III
Case 5	36	Female	24.2	No	No	Unstageable
Case 6	17	Female	18.8	No	No	IIB

**Table 2:** Participants’ wound area size as measured at each visit.

Participant n.	Visit 1 Wound Area	Visit 2 Wound Area *	Visit 3 Wound Area
Case 1	23.4 mm^2^	27.4 mm^2^	22.7 mm^2^ (−17.6%)
Case 2	59.0 mm^2^	78.5 mm^2^	45.2 mm^2^ (−42.4%)
Case 3	6.9 mm^2^	29.6 mm^2^	10.7 mm^2^ (−63.9%)
Case 4	75 mm^2^	160 mm^2^	136 mm^2^ (−15%)
Case 5	39.8 mm^2^	50.7 mm^2^	70.4 mm^2^ (+38.9%)
Case 6	47.7 mm^2^	48.1 mm^2^	45.5 mm^2^ (−5.4)

* Visit 2 wound areas refers to measurement post-surgery.

**Table 3:** Participants’ cell numbers in exudates at different visits.

Exudate Cell Number (in 100 μL)			
	Visit 1	Visit 2	Visit 3 *
Case 1	12,352	58,800	1,494,000 (2540%)
Case 2	21,851	766,451	540,800 (70%)
Case 3	5468	12,404	5125 (41.3%)
Case 4	210,370	374,375	137,666 (36.8%)
Case 5	666,000	232,142	403,571 (173.8%)
Case 6	20,833	6,666	150,000 (2250%)

* Percentages refer to cell number changes from Visit 2 to Visit 3.

**Table 4:** Study protocol followed at each visit.

Visit 1 Nail Surgery Assessment Appointment	Visit 2 Surgery Day	Visit 3 24 h Post-Surgery Follow Up
- Recruitment and consent. - Collection of demographic and clinical data. - Collection of Exudate.	- Collection of last Melolin applied 24 h before surgery. - Collection of Exudate (after nail removal, before phenol application).	- Collection of Atrauman and Melolin applied after surgery. - Collection of exudates.
- Application of Melolin. - Subject advised to redress daily with Melolin.	- Application of Atrauman and Melolin. - Subject advised to keep the dressings in place until Visit 3.	- Application of Mepore. - Subject discharged.

**Table 5:** Onychocryptosis classification adopted to stage participants’ IGTN at Visit 1.

Stage	Description
I	Erythema, slight oedema, and pain. Nail fold does not exceed the limits of the nail plate.
II	A. Increased pain, oedema, erythema, hyperesthesia, serum drainage, and/or infection. Nail fold exceeds the nail plate < 3 mm.
	B. Increased pain, oedema, erythema, hyperesthesia, serum drainage, and/or infection. Nail fold exceeds the nail plate > 3 mm.
III	Granulation tissue and chronic hypertrophy of the nail fold. Granulomatous or hypertrophic tissue widely covers the lateral nail plate.
IV	Serious chronic deformity of the toenail, both nail folds and distal fold. Hypertrophic tissue completely covers lateral, medial and distal nail plates.

## Data Availability

Raw data are available in the University of Brighton Open Repository.
